# Comparison of gadolinium-based contrast agents for MR cholangiography in saline, blood and bile: a phantom study

**DOI:** 10.1186/s41747-023-00331-2

**Published:** 2023-04-24

**Authors:** Johannes M. Froehlich, Leen Moussa, Natalie Guirguis, Andreas Gutzeit, David Wu, Sabine Sartoretti-Schefer, Dow-Mu Koh, Orpheus Kolokythas, Simon Matoori

**Affiliations:** 1 Clinical Research Group, Klus Apotheke Zurich, Zurich, Switzerland; 2 Faculté de Pharmacie, Université de Montréal, 2940 Chemin de Polytechnique, Montreal, QC H3T 1J4 Canada; 3 Institute of Radiology and Nuclear Medicine and Breast Center St. Anna, Hirslanden Klinik St. Anna, Lucerne, Switzerland; 4 Department of Health Sciences and Medicine, Universität Luzern, Frohburgstrasse 3, 6002 Lucerne, Switzerland; 5 Laboratory for Cell and Tissue Engineering, Harvard John A. Paulson School of Engineering and Applied Sciences, Harvard University, Cambridge, MA 02138 USA; 6 Wyss Institute for Biologically Inspired Engineering, Harvard University, Cambridge, MA 02138 USA; 7 Department of Oral Medicine, Infection and Immunity, Harvard School of Dental Medicine, Boston, MA 02115 USA; 8 Institute of Radiology, Kantonsspital Winterthur, Brauerstrasse 15, 8401 Winterthur, Switzerland; 9 Cancer Research UK Clinical Magnetic Resonance Research Group, Institute of Cancer Research, Sutton, Surrey UK; 10 Department of Radiology, University of Washington Medical Center, Seattle, WA USA

**Keywords:** Magnetic resonance imaging, Contrast media, Gadolinium, Bile, Bile ducts

## Abstract

**Background:**

We compared T1- and T2-weighted signal intensities of liver-specific (gadoxetate, gadobenate) and non-specific (gadoterate) gadolinium contrast agents (CAs) in a bile phantom.

**Methods:**

In a phantom study, gadoxetate, gadobenate, and gadoterate were diluted in saline, blood, and bile at different concentrations (0, 0.25, 0.5. 1, 2.5, 5, 10, and 25 mM) and imaged in a 3-T magnetic resonance imaging (MRI) system using T1- and T2-weighted sequences. The maximum signal intensities of CAs were compared for each sequence separately and across all T1-weighted sequences using one-way ANOVA.

**Results:**

Using T1-weighted sequences, CA concentration-dependent signal intensity increase was followed by decrease due to T2* effects. Comparing CAs for each sequence in bile yielded higher maximum signal intensities with gadobenate than gadoxetate and gadoterate using T1-weighted spin-echo (*p* < 0.010), multiecho gradient- and spin-echo (*p* < 0.001), and T1-weighted high-resolution isotropic volume excitation (eTHRIVE) sequences (*p* < 0.010). Comparing across all T1-weighted sequences in the bile phantom, gadobenate imaged using T1-weighted turbo field-echo (TFE) sequence showed the highest signal intensity, significantly higher than that using other CAs agents or sequences (*p* < 0.004) except for gadobenate and gadoxetate evaluated with three-dimensional multiecho fast field-echo (3D-mFFE) and gadoxetate with T1-weighted TFE sequence (*p* > 0.141). Signal reduction with CA concentration-dependent decrease was observed on T2-weighted images.

**Conclusion:**

In this bile phantom study of gadolinium-based CA, gadobenate and gadoxetate showed high signal intensity with T1-weighted TFE and 3D-mFFE sequences, which supports their potential utility for contrast-enhanced hepatobiliary MRI.

**Key points:**

• Contrast-enhanced magnetic resonance (MR) cholangiography depends on contrast agent type, kinetics, and concentration in bile,

• We compared signal intensities of three contrast agents in a bile phantom study.

• Gadobenate, gadoxetate, and gadoterate demonstrated different signal intensities at identical concentrations.

• Gadoxetate and gadobenate showed high signal intensities on T1-weighted MR sequences.

**Supplementary Information:**

The online version contains supplementary material available at 10.1186/s41747-023-00331-2.

## Background

Magnetic resonance (MR) cholangiography has gained broad acceptance for the evaluation of multiple biliary pathologies [1]. It is commonly used for the detection of choledocholithiasis, benign and malignant strictures, primary sclerosing or other forms of cholangitis, as well as for preoperative planning [1].

While MR cholangiography is often performed without contrast enhancement, there is an increasing interest in the use of hepatobiliary contrast agents such gadoxetate and gadobenate [1–4]. These contrast agents are linear, ionic, and hepatocyte-selective T1 weighted gadolinium-based MR agents with a relatively high relaxivity [5, 6]. While the other linear MR contrast agents gadopentetate, gadodiamide, gadoversetamide were suspended due to dechelation (transmetallation)-associated safety concerns by the European Medicine’s Agency in 2017, gadoxetate and gadobenate remained on the market in Europe with a restricted indication for hepatobiliary MR [7]. In the United States, the Food and Drug Administration gave warning labels due to dechelation to all gadolinium-based contrast agents. In the Food and Drug Administration -approved label (as of 01/2023), gadoxetate is indicated for liver MR imaging (MRI), while gadobenate is indicated for MRI of the central nervous system and MR angiography. Gadoxetate and gadobenate are administered intravenously, taken up by hepatocytes (about 50% of gadoxetate dose and 3–5% of gadobenate dose) and excreted partly through the biliary system [5, 6]. Both agents are actively transported into hepatocytes by organic anion transporter polypeptide B1 subtypes and subsequently excreted into biliary canaliculi by the canalicular multispecific organic anion transporter [5, 6, 8]. Non-liver specific contrast agents such as gadoterate lead to vascular and interstitial contrast enhancement. Using gadoxetate and gadobenate, visualization of the biliary tree begins 10 to 100 min after injection and lasts for 1–4 h [1, 8]. These liver-specific contrast agents can provide both anatomical and functional information of hepatocytes and bile ducts. While the most common indication is the detection and characterization of focal liver lesions, contrast enhancement in the parenchyma and biliary system has been used to grade liver cirrhosis, for prognosis of acute-on-chronic liver failure, to detect bile ducts leaks after surgery, and to assess the biliary anatomy of liver donors [1, 9–16].

With the growing importance of contrast-enhanced MR cholangiography, there is a clinical need to understand which contrast agent has the highest signal intensity in bile. However, to the best of our knowledge, contrast agent signal intensities in bile are currently not reported in the literature. This gap in the literature complicates evidence-based contrast agent selection for contrast-enhanced MR cholangiography. Therefore, the focus of our phantom study was to determine and compare the signal intensities of two hepatobiliary contrast agents (gadoxetate, gadobenate) and an extracellular agent (gadoterate) in bile, blood, and saline using clinically used MR pulse sequences. We hypothesize that these findings will support evidence-based selection of contrast agents and pulse sequences for contrast-enhanced MR cholangiography.

## Methods

### Saline, blood, and bile phantoms

No Institutional Review Board approval or Informed Consent was needed for this phantom study.

**Fig. 1:**
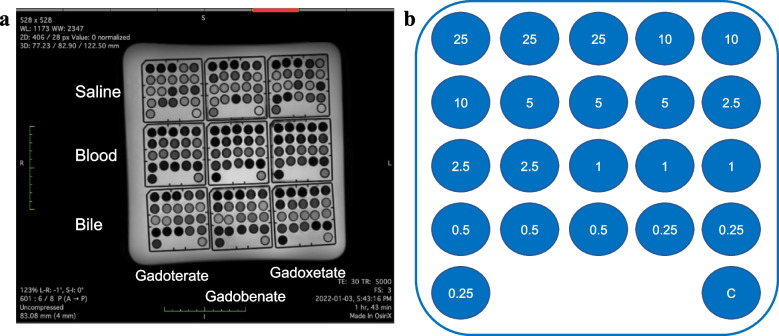
Experimental set-up. The vials containing contrast agents gadoterate (left column), gadobenate (middle column), and gadoxetate (right column) in saline (top row), blood (middle row), and bile (lower row) were placed in a sample holder and submerged in water at room temperature. The samples were then imaged using different sequences (here: fluid-attenuated inversion-recovery sequence) (**a**). The concentration of the contrast agents ranged from 0.25 to 25 mM, and each sample contained a control vial on the lower right with saline instead of the contrast agent (**b**)

The contrast agents gadoxetate (Primovist/Eovist, Bayer, Leverkusen, Germany), gadobenate (Multihance, Bracco, Milan, Italy), and gadoterate (Dotarem, Guerbet, Villepinte, France) were diluted in saline (saline phantom), anticoagulated ox (sex: male; age: 2 years; origin: Switzerland) blood (blood phantom) and ox (sex: male; age: 2 years; origin: Switzerland) bile (bile phantom) at different concentrations (0, 0.25, 0.5. 1, 2.5, 5, 10, 25 mM). They were imaged in a 3-T MR system (Ingenia, Philips Healthcare, Best, the Netherlands) using a phased-array 16-channel coil with the following experimental setting: 2.0 mL of solution containing at least 90 volume% of sodium chloride 0.9%, blood, or bile, was put in 2 mL polypropylene tubes (diameter 10 mm, surface area 79 mm^2^) in a sample holder (75 mm × 75 mm, capacity 25 tubes, Nalgene, Rochester, New York, NY, USA) submerged in water at room temperature (Fig. 1, Supplementary Fig. S1). Each condition was prepared in triplicates (*n* = 3). The images were analyzed using OsiriX DICOM viewer (Pixmeo SARL, Bernex, Switzerland). A round region-of-interest of approximately 30 mm^2^ was placed on each  vial. The median signal intensity of the region-of-interest was reported.

### MR sequences

The pulse sequences were based on slightly modified standard clinical sequences provided by Philips for the 3-T MR system and included a T1-weighted spin echo (SE) sequence, a T1-weighted turbo field-echo (TFE) sequence, a three-dimensional (3D) multiecho fast field-echo (mFFE) sequence, a multiecho gradient- and spin-echo (mGraSE) sequence, a short tau inversion-recovery (STIR) sequence, a 3D T1-weighted high-resolution isotropic volume excitation (eTHRIVE) sequence, a two-dimensional (2D) MIXED sequence, a T2-weighted turbo SE (TSE) sequence, and a fluid attenuated inversion-recovery (FLAIR) sequence. The sequence parameters are reported in Table 1.

**Table 1:** Pulse sequence parameters employed in this study

Sequence	Repetition time (ms)	Echo time (ms)	Flip angle (degrees)
T1-weighted spin-echo	600–700	10	70
T1-weighted turbo field-echo	“shortest”	“shortest”	8
3D multiecho fast field-echo	“shortest”	4.6	20
Multiecho gradient- and spin-echo	“shortest”	“shortest”	90
Short tau inversion-recovery	5,000	30	90
T1-weighted high-resolution isotropic volume excitation	“shortest”	“shortest”	10
2D MIXED	920 (spin-echo) 2,300 (inversion-recovery)	“shortest”	90
T2-weighted turbo spin-echo	3,000	80	90
Fluid-attenuated inversion-recovery	11,000	125	90

*2D* Two-dimensional, *3D* Three-dimensional

### Statistical analysis

The statistical calculations were carried out by SigmaPlot version 13.0 (SPSS Inc, Chicago, IL). The maximum signal intensity (*i.e.*, mean of three highest signal intensities per condition) of the three contrast agents was calculated. First, the maximum signal intensity of the three contrast agents was compared for each pulse sequence separately using one-way ANOVA followed by a Tukey's post-hoc test. Second, the maximum signal intensities were compared over all T1-weighted sequences in bile (T1-weighted SE, T1-weighted TFE, 3D-mFFE, mGraSE, STIR, eTHRIVE) using a one-way ANOVA followed by a Tukey's post-hoc test. A *p*-value of < 0.050 was deemed statistically significant. In the figures, significant differences between the three contrast agents are presented as follows:

* = significantly higher maximum signal intensity of the contrast agent than both other contrast agents; +  = significantly higher maximum signal intensity than the contrast agent with the lowest maximum signal intensity;

* or +  = *p* < 0.050;

** or +  +  = *p* < 0.010;

*** or +  +  +  = *p* < 0.001.

## Results

### T1-weighted pulse sequences

**Fig. 2:**
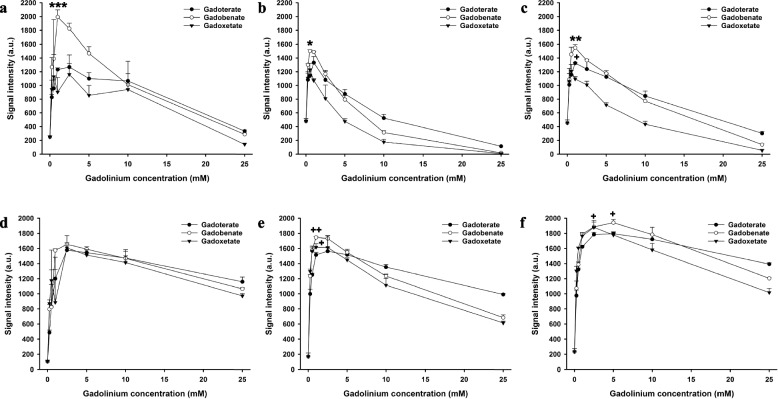
Signal intensities of gadoterate, gadobenate, and gadoxetate in saline and biological fluids on T1-weighted spin-echo and turbo field-echo MRI. Signal intensities of gadoterate, gadobenate, and gadoxetate at different concentrations in saline (**a**), blood (**b**), and bile (**c**) on T1-weighted spin echo MRI. Signal intensities of gadoterate, gadobenate, and gadoxetate at different concentrations in saline (**d**), blood (**e**), and bile (**f**) on T1-weighted turbo field-echo sequence. * = significantly higher maximum signal intensity of the contrast agent than both other contrast agents; +  = significantly higher maximum signal intensity than the contrast agent with the lowest maximum signal intensity. * or +  = *p* < 0.050, ** or +  +  = *p* < 0.010; *** or +  +  +  = *p* < 0.001

**Fig. 3:**
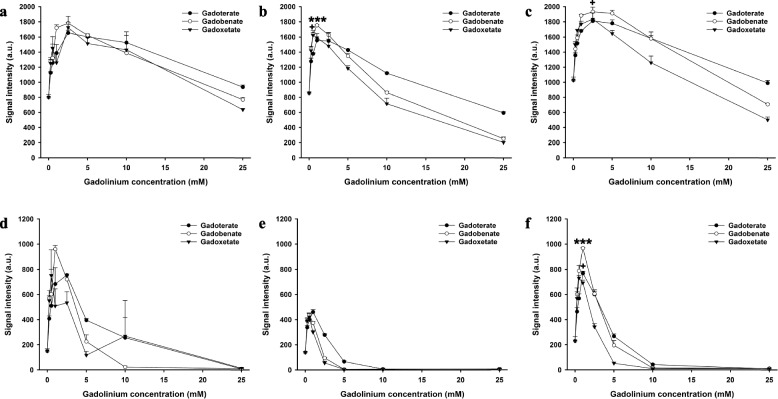
Signal intensities of gadoterate, gadobenate, and gadoxetate in saline and biological fluids on three-dimensional multiecho fast field-echo (3D mFFE) and multiecho gradient- and spin-echo (mGraSE) sequences. Signal intensities of gadoterate, gadobenate, and gadoxetate at different concentrations in saline (**a**), blood (**b**), and bile (**c**) on 3D-mFFE sequence. Signal intensities of gadoterate, gadobenate, and gadoxetate at different concentrations in saline (**d**), blood (**e**), and bile (**f**) on mGraSE sequence. * = significantly higher maximum signal intensity of the contrast agent than both other contrast agents; +  = significantly higher maximum signal intensity than the contrast agent with the lowest maximum signal intensity. * or +  = *p* < 0.050, ** or +  +  = *p* < 0.010; *** or +  +  +  = *p* < 0.001

**Fig. 4:**
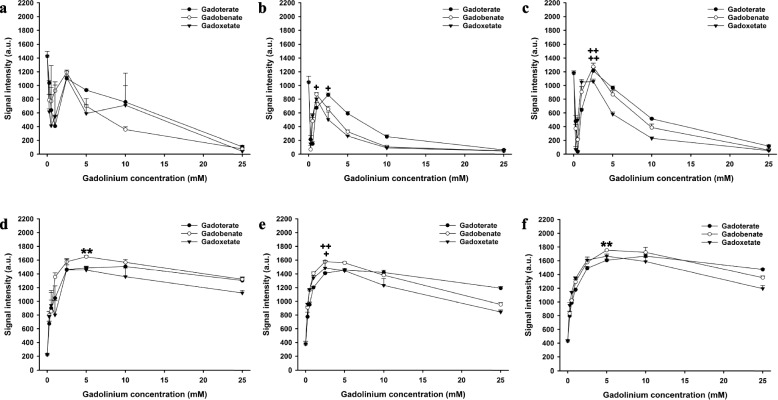
Signal intensities of gadoterate, gadobenate, and gadoxetate in saline and biological fluids on short tau inversion-recovery (STIR) and T1-weighted high-resolution isotropic volume excitation (eTHRIVE) sequences. Signal intensities of gadoterate, gadobenate, and gadoxetate at different concentrations in saline (**a**), blood (**b**), and bile (**c**) on STIR sequence. Signal intensities of gadoterate, gadobenate, and gadoxetate at different concentrations in saline (**d**), blood (**e**), and bile (**f**) on e-THRIVE sequence. * = significantly higher maximum signal intensity of the contrast agent than both other contrast agents; +  = significantly higher maximum signal intensity than the contrast agent with the lowest maximum signal intensity. * or +  = *p* < 0.050, ** or +  +  = *p* < 0.010; *** or +  +  +  = *p* < 0.001

**Fig. 5:**
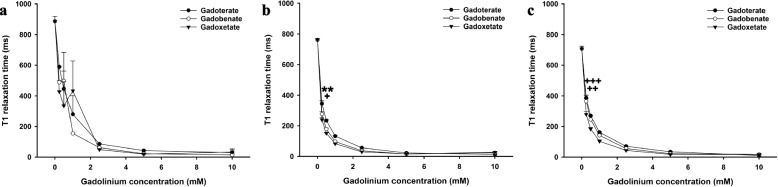
T1 relaxation time of gadoterate, gadobenate, and gadoxetate on 2D MIXED sequence. T1 relaxation time of gadoterate, gadobenate, and gadoxetate at different concentrations in saline (**a**), blood (**b**), and bile (**c**) on 2D MIXED sequence. * = significantly higher maximum signal intensity of the contrast agent than both other contrast agents; +  = significantly higher maximum signal intensity than the contrast agent with the lowest maximum signal intensity. * or +  = *p* < 0.050, ** or +  +  = *p* < 0.010; *** or +  +  +  = *p* < 0.001

On T1-weighted pulse sequences, a contrast agent concentration-dependent increase in signal intensity was followed by a plateau and a subsequent decrease due to T2 and T2* effects (Figs. 2, 3, 4, and 5 Supplementary Figs. S2−S4, Supplementary Table S1).

First, we compared the maximum signal intensities of the three contrast agents for each pulse sequence. In saline, the maximum intensity of gadobenate was significantly higher than gadoterate and gadoxetate with the T1-weighted SE (*p* < 0.001) and the eTHRIVE sequence (*p* < 0.010). No significant differences were observed between the maximum signal intensities of the contrast agents with the other sequences (*p* > 0.054). There was no significant difference in maximum relaxation time between the contrast agents with the 2D MIXED sequence (*p* = 0.516).

In blood, the maximum intensity of gadobenate and gadoxetate were higher than gadoterate with the T1-weighted SE sequence (*p* < 0.050). The maximum intensity of gadobenate and gadoxetate were higher than gadoterate with the T1-weighted TFE sequence (*p* < 0.050). The maximum intensity of gadobenate was higher than gadoxetate and gadoterate with the 3D mFFE sequence (*p* < 0.001). The maximum intensity of gadobenate and gadoterate were higher than gadoxetate with the STIR sequence (*p* < 0.050). The maximum intensity of gadobenate and gadoxetate were higher than gadoterate with the eTHRIVE sequence (*p* < 0.050). No significant differences were observed between the maximum signal intensities of the contrast agents with the mGraSE sequence (*p* = 0.323).

In bile, the maximum intensity of gadobenate was higher than gadoxetate and gadoterate with the T1-weighted SE sequence (*p* < 0.010), and the maximum intensity of gadoterate was higher than gadoxetate (*p* < 0.050). The maximum intensity of gadobenate and gadoxetate were higher than gadoterate with the T1-weigthed TFE sequence (*p* < 0.050). The maximum intensity of gadobenate was higher than gadoterate with the 3D-mFFE sequence (*p* < 0.050), and the difference between gadobenate and gadoxetate was not significantly different (*p* = 0.098). The maximum intensity of gadobenate was higher than gadoterate and gadoxetate with the mGraSE sequence (*p* < 0.001), and the signal intensity of gadoterate was higher than gadoxetate with this sequence (*p* < 0.050). The maximum intensities of gadobenate and gadoterate were higher than gadoxetate with the STIR sequence (*p* < 0.010). The maximum intensity of gadobenate was higher than gadoxetate and gadoterate with the eTHRIVE sequence (*p* < 0.010). The maximum relaxation time of gadobenate and gadoterate were higher than gadoxetate with the 2D MIXED sequence (*p* < 0.010).

Second, we compared the maximum signal intensities of all contrast agents across T1-weighted SE, T1-weighted TFE, eTHRIVE, GRase, and STIR sequences in bile. The highest maximum signal intensity was observed for gadobenate with the T1-weighted TFE pulse sequence, which was higher than the intensities of all contrast agents with the eTHRIVE, GRase, T1-weighted SE, and STIR sequences, and the maximum intensity of gadoterate with the T1-weighted TFE and 3D-mFFE sequences (*p* < 0.004). However, in this comparison across all sequences, the maximum signal intensity of gadobenate with the T1-weighted TFE sequence was neither significantly higher than maximum signal intensity of gadoxetate with the T1-weighted TFE sequence nor significantly higher than maximum signal intensity of gadoxetate and gadobenate with the 3D-mFFE sequence (*p* > 0.141).

To compare our results to the literature (see below), we also compared the signal intensities at a fixed contrast agent concentration of 0.25 mM in saline and blood on T1-weighted TFE MRI. In saline, we observed a higher signal intensity of gadobenate than gadoterate (*p* < 0.001) a higher signal intensity of gadoxetate than gadoterate (*p* < 0.001), and no significant difference between gadobenate and gadoxetate (*p* = 0.188) on T1-weighted TFE MRI. In blood, where we observed a higher signal intensity of gadobenate than gadoterate (*p* < 0.001) a higher signal intensity of gadoxetate than gadoterate (*p* = 0.001), and no significant difference between gadobenate and gadoxetate (*p* = 0.229) on T1-weighted TFE MRI.

### T2-weighted pulse sequences

**Fig. 6:**
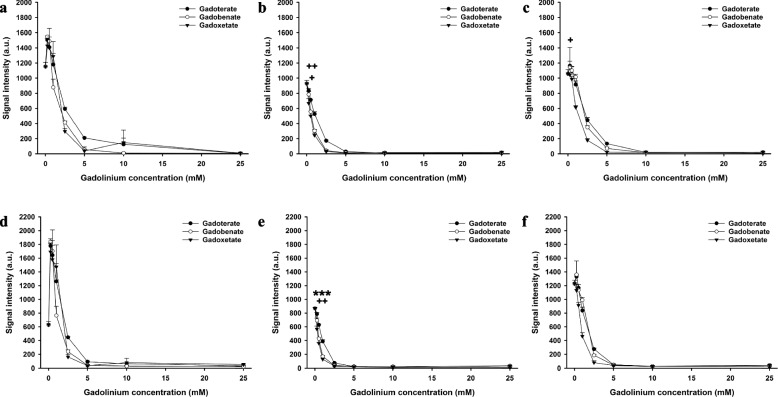
Signal intensities of gadoterate, gadobenate, and gadoxetate in saline and biological fluids on T2-weighted TSE and fluid-attenuated (FLAIR) sequence. Signal intensities of gadoterate, gadobenate, and gadoxetate at different concentrations in saline (**a**), blood (**b**), and bile (**c**) on T2-weighted turbo spin-echo MRI. Signal intensities of gadoterate, gadobenate, and gadoxetate at different concentrations in saline (**d**), blood (**e**), and bile (**f**) on FLAIR sequence. * = significantly higher maximum signal intensity of the contrast agent than both other contrast agents; +  = significantly higher maximum signal intensity than the contrast agent with the lowest maximum signal intensity. * or +  = *p* < 0.050, ** or +  +  = *p* < 0.010; *** or +  +  +  = *p* < 0.001

In T2-weighted pulse sequences, signal intensities decreased with increasing contrast agent concentration (Fig. 6, Supplementary Fig. S5, Supplementary Table S1).

In saline, no significant differences among maximum contrast agent signal intensities were observed in the T2-weighted TSE and the FLAIR sequences (*p* > 0.111).

In blood, gadobenate and gadoterate were significantly higher maximum contrast agent signal intensities than gadoxetate with the T2-weighted TSE sequence (*p* < 0.010). The maximum contrast agent signal intensity of gadoterate was higher than gadoxetate and gadobenate with the FLAIR sequence (*p* < 0.001), and maximum signal intensity of gadobenate was higher than gadoxetate (*p* < 0.010).

In bile, maximum signal intensity of gadobenate was higher than gadoxetate with the T2-weighted TSE sequence (*p* < 0.050). No significant differences in maximum signal intensity were observed with the FLAIR sequence (*p* = 0.123). The results of intra-sequence comparisons of maximum signal intensities of contrast agents are summarized in Supplementary Table S1.

## Discussion

In this study, we investigated the signal intensity of three contrast agents in a bile phantom and observed differences between contrast agents, contrast agent concentrations, and pulse sequences. The maximum signal intensity of gadobenate with the T1-weighted SE sequence was higher than all other contrast agents and conditions except for gadobenate and gadoxetate with 3D mFFE and gadoxetate with the T1-weighted SE sequence (*p* < 0.004), which confirms the usefulness of gadobenate and gadoxetate for contrast-enhanced hepatobiliary MRI. To the best of our knowledge, this is the first study to determine and compare signal intensities of MRI contrast agents in a bile phantom.

In T1-weighted pulse sequences, we observed a concentration-dependent increase in signal intensity followed by a decrease due to T2* effects which is in accordance with the literature [17]. Pulse sequence- and contrast agent-dependent differences were also observed. Comparing contrast agents for each pulse sequences separately yielded higher maximum signal intensities of gadobenate than gadoxetate and gadoterate with T1-weighted SE (*p* < 0.010), mGraSE (*p* < 0.001), and eTHRIVE sequences (*p* < 0.010) in bile.

In bile, we observed that the maximum signal intensity of gadobenate was higher than all other contrast agents and conditions except for gadobenate and gadoxetate with 3D-mFFE and gadoxetate with the T1-weighted SE sequence (*p* < 0.004). This finding complements recent reports which pointed to the usefulness of gadobenate-enhanced biliary imaging using a T1-weighted gradient echo pulse sequence to stage liver cirrhosis and predict outcomes in acute-on-chronic liver failure [9, 10]. Nevertheless, more studies are needed to determine whether gadobenate is the most suitable contrast agent for biliary imaging. In this study, we saw a strong contrast agent concentration dependence in signal intensities. Therefore, the contrast agent concentration in the biliary canaliculi will be an important factor determining the signal intensity. The contrast agent concentration is likely related to the uptake of the contrast agent into the liver, which is higher for gadoxetate (about 50%) than gadobenate (3–5%) [5, 6], and the administered dose, which is lower for gadoxetate (0.025 mmol/kg body weight) than gadobenate (0.1 mmol/kg body weight). To contextualize our findings, it is therefore important to note that multiple factors impact the observed signal intensity in MR cholangiography in the clinical setting: the inherent relaxivity of contrast agents in bile and the employed MR sequence (*i.e.*, the focus of our study), and the concentration of the contrast agent in the bile canaliculi. The latter depends on the administered dose, and the amount and kinetics of contrast agent uptake into hepatocytes and excretion into bile canaliculi. This is ultimately also related to the functionality of the organic anion transporting polypeptides (OATP) uptake and multidrug resistance-associated protein 2 (MRP2) efflux transporters.

Previously, the relaxivities of gadoxetate, gadobenate, and gadoterate were compared in different media: Rohrer et al. [18] found that the relaxivity of gadobenate was similar to gadoxetate, and that these two contrast agents exhibited higher relaxivities than gadoterate in water, plasma, and blood with a T1-weighted TSE sequence at 1.5-T (blood) and 3-T (water, plasma) MRI. Moreover, Goetschi et al. [19] found higher relaxivity of gadoxetate compared with gadoterate in saline. To compare our results to these studies, we focused on a specific contrast agent concentration of 0.25 mM which is most comparable to other literature reports. In saline, we observed a higher signal intensity of gadobenate than gadoterate (*p* < 0.001) a higher signal intensity of gadoxetate than gadoterate (*p* < 0.001), and no significant difference between gadobenate and gadoxetate (*p* = 0.188) on T1-weighted TFE MRI. Analogous results were observed in blood, where we saw a higher signal intensity of gadobenate than gadoterate (*p* < 0.001) a higher signal intensity of gadoxetate than gadoterate (*p* = 0.001), and no significant difference between gadobenate and gadoxetate (*p* = 0.229) on T1-weighted TFE MRI. Therefore, our results are in agreement with the literature [18, 19].

This study has several limitations. The main limitation is that typical contrast agent concentrations of gadoxetate and gadobenate in bile ducts could not be retrieved from the literature. Therefore, it is difficult to estimate which contrast agent will have the highest signal intensity in the clinical setting where the administered dose, degree and kinetics of uptake into hepatocytes, and kinetics of contrast agent secretion into the biliary system determine the concentration of contrast agent in bile. As the uptake into hepatocytes and the secretion into bile are mediated by OATP uptake transporters and MRP2 efflux transporters, respectively, these processes are highly time-dependent, impacted by co-morbidities (*e.g.*, liver disease) and the use of medication that compete with the contrast agents for the hepatocyte uptake transporters (*e.g.*, statins) [20, 21]. Therefore, more clinical studies are warranted to study potential effects of genetics, race/ethnicity, sex and gender differences, co-morbidities, and medication use on biliary contrast agent concentrations. While it would be highly invasive to determine gadoxetate or gadobenate concentrations in bile ducts by biopsy, MRI signal intensities or relaxation times of these contrast agents in bile could be useful to determine these concentrations. Position effects due to field inhomogeneities cannot be excluded despite the use of a phased-array 1- channel coil. Furthermore, only a 3-T scanner was used and other field strengths were not investigated. The biologic fluids originated from ox as human fluids were not available, and were stored at -25 °C prior to use. Moreover, slight changes in composition of biological fluids over the time of the experiment cannot be excluded despite temperature control and the use of an anticoagulant. Potential changes in composition will only affect inter-sequence comparisons and not intra-sequence comparisons, as all conditions were recorded at the same time in each sequence.

In this bile phantom study of gadolinium-based contrast agents, gadobenate and gadoxetate showed high signals with T1-weighted TFE and 3D mFFE pulse sequences, which supports their potential suitability for contrast-enhanced hepatobiliary MRI. Our study also shows a strong concentration dependence of the contrast agent signal intensity in bile. In the clinical setting, the contrast agent concentration in bile depends on the administered dose, liver uptake, and secretion into bile. Our findings indicate that more clinical studies are warranted to compare biliary contrast agent enhancement patterns in the bile ducts of patients. As our study quantifies the signal intensity of the two contrast agents in bile in a controlled *in vitro* environment, it could be a useful reference for future clinical studies that quantify contrast agent concentrations in the biliary system to investigate hepatocyte function and hepatobiliary diseases.

## Supplementary Information

**Additional file 1: Supplementary Figure S1. **Bile phantom set up. Ox bile and contrast agents were mixed in 2 mL polypropylenetubes (bile phantom, **a**) and placed in a sample holder **b**. **Supplementary Figure S2. **Signal intensities of gadoterate, gadobenate, and gadoxetate in saline and biological fluids on T1-weighted SE and TFE MRI. Signal intensities of gadoterate, gadobenate, and gadoxetate at different concentrations in saline (**a**), blood (**b**), and bile (**c**) on T1-weighted spin echo MRI. Signal intensities ofgadoterate, gadobenate, and gadoxetate at different concentrations in saline (**d**), blood (**e**), and bile (**f**) on T1-weighted turbo field echo MRI. *: significantly higher maximum signal intensity of the contrast agent than both other contrast agents; +: significantly higher maximum signal intensity than the contrast agent with the lowest maximum signal intensity. * or +: *p* < 0.05,** or ++: *p* < 0.01; *** or +++: *p *< 0.001. The data are extracted from Figure 2. **Supplementary Figure S3. **Signal intensities of gadoterate, gadobenate, and gadoxetate in saline and biological fluids on 3D-mFFE and mGraSE MRI. Signal intensities of gadoterate, gadobenate, and gadoxetate at different concentrations in saline (**a**), blood (**b**), and bile (**c**) on 3D-mFFE MRI. Signal intensities of gadoterate, gadobenate, and gadoxetate at different concentrations in saline (**d**), blood (**e**), and bile (**f**) on mGraSE MRI. The data are extracted from Figure 3. **Supplementary Figure S4. **Signal intensities of gadoterate, gadobenate, and gadoxetate in saline and biological fluids on STIR and eTHRIVE MRI. Signal intensities of gadoterate, gadobenate, and gadoxetate at different concentrations in saline (**a**), blood (**b**), and bile (**c**) on STIR MRI. Signal intensities of gadoterate, gadobenate, and gadoxetateat different concentrations in saline (**d**), blood (**e**), and bile (**f**) on E-THRIVE MRI. The data are extracted from Figure 4. **Supplementary Figure S5. **Signal intensities of gadoterate, gadobenate, and gadoxetate in saline and biological fluids on T2-weighted TSE and FLAIR MRI. Signal intensities of gadoterate, gadobenate, and gadoxetate at different concentrations in saline (**a**), blood (**b**), and bile (**c**) on T2-weighted TSE MRI. Signal intensities of gadoterate, gadobenate, and gadoxetate at different concentrations in saline (**d**), blood (**e**), and bile (**f**) on FLAIR MRI. The data are extracted from Figure 6. **Supplementary Table S1. **Summary table of main results in the comparisons of maximum signal intensities of contrast agents.

## Data Availability

The data and materials are available from the authors upon request.

## Abbreviations

2D: Two-dimensional
3D: Three-dimensional
eTHRIVE: T1-weighted high-resolution isotropic volume excitation
FLAIR: Fluid-attenuated inversion-recovery
mFFE: Multiecho fast field-echo
mGraSE: Multiecho gradient- and spin-echo
MR: Magnetic resonance
MRI: Magnetic resonance imaging
MRP2: Multidrug resistance-associated protein 2
OATP: Organic anion transporting polypeptides
SE: Spin-echo
STIR: Short tau inversion-recovery
TFE: Turbo field-echo
TSE: Turbo spin-echo
