# Supplementary material for: Comparison of gadolinium-based contrast agents for MR cholangiography in saline, blood and bile: a phantom study
Source: Eur Radiol Exp. 2023 Apr 24;7:21. doi: 10.1186/s41747-023-00331-2 (PMC10126166; doi:10.1186/s41747-023-00331-2)
Supplement: Supplementary file 1 — Additional file 1: Supplementary Figure S1. Bile phantom set up. Ox bile and contrast agents were mixed in 2 mL polypropylenetubes (bile phantom, a) and placed in a sample holder b. Supplementary Figure S2. Signal intensities of gadoterate, gadobenate, and gadoxetate in saline and biological fluids on T1-weighted SE and TFE MRI. Signal intensities of gadoterate, gadobenate, and gadoxetate at different concentrations in saline (a), blood (b), and bile (c) on T1-weighted spin echo MRI. Signal intensities ofgadoterate, gadobenate, and gadoxetate at different concentrations in saline (d), blood (e), and bile (f) on T1-weighted turbo field echo MRI. *: significantly higher maximum signal intensity of the contrast agent than both other contrast agents; +: significantly higher maximum signal intensity than the contrast agent with the lowest maximum signal intensity. * or +: p < 0.05,** or ++: p < 0.01; *** or +++: p < 0.001. The data are extracted from Figure 2. Supplementary Figure S3. Signal intensities of gadoterate, gadobenate, and gadoxetate in saline and biological fluids on 3D-mFFE and mGraSE MRI. Signal intensities of gadoterate, gadobenate, and gadoxetate at different concentrations in saline (a), blood (b), and bile (c) on 3D-mFFE MRI. Signal intensities of gadoterate, gadobenate, and gadoxetate at different concentrations in saline (d), blood (e), and bile (f) on mGraSE MRI. The data are extracted from Figure 3. Supplementary Figure S4. Signal intensities of gadoterate, gadobenate, and gadoxetate in saline and biological fluids on STIR and eTHRIVE MRI. Signal intensities of gadoterate, gadobenate, and gadoxetate at different concentrations in saline (a), blood (b), and bile (c) on STIR MRI. Signal intensities of gadoterate, gadobenate, and gadoxetateat different concentrations in saline (d), blood (e), and bile (f) on E-THRIVE MRI. The data are extracted from Figure 4. Supplementary Figure S5. Signal intensities of gadoterate, gadobenate, and gadoxetate in salin [file 41747_2023_331_MOESM1_ESM.pdf]

ELECTRONIC SUPPLEMENTARY MATERIAL

Comparison of gadolinium-based contrast agents for MR cholangiography in saline, blood and bile: a phantom study

Eur Radiol Exp (2023) Froehlich JM, Moussa L, Guirguis N et al.

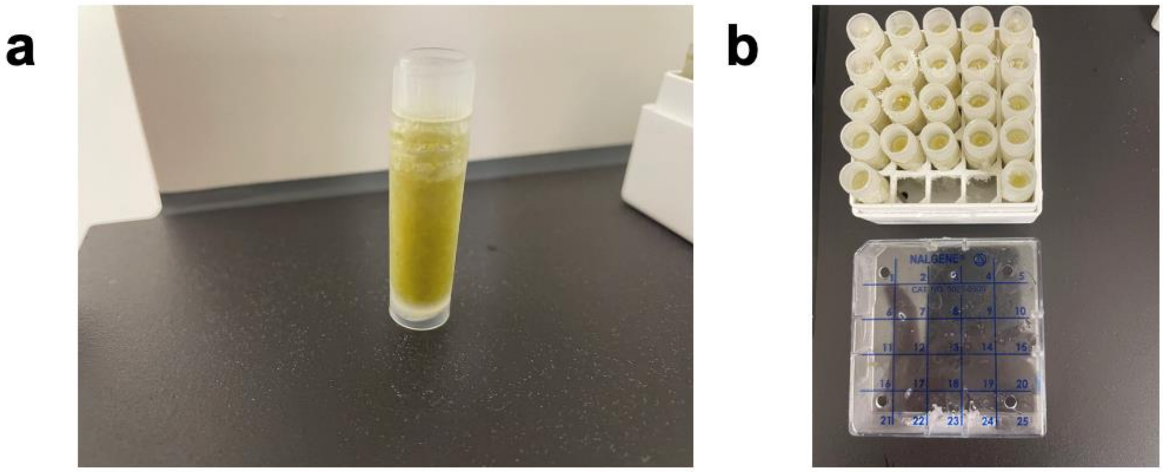

Supplementary Figure S1. Bile phantom set up. Ox bile and contrast agents were mixed in 2 mL polypropylene tubes (bile phantom, a) and placed in a sample holder (b).

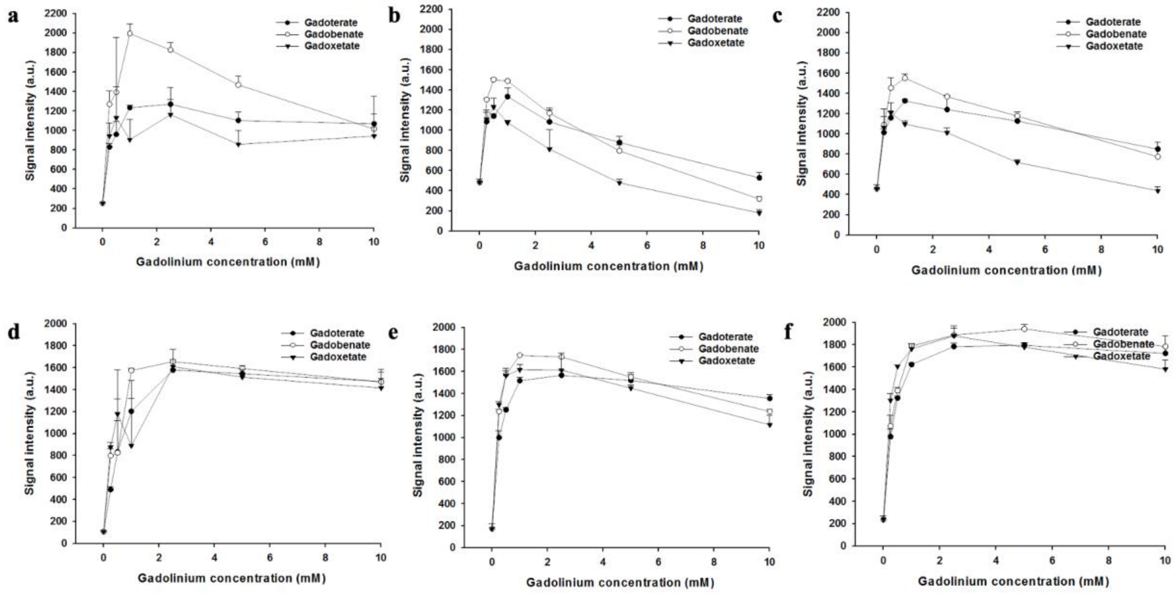

Supplementary Figure S2. Signal intensities of gadoterate, gadobenate, and gadoxetate in saline and biological fluids on T1-weighted SE and TFE MRI. Signal intensities of gadoterate, gadobenate, and gadoxetate at different concentrations in saline (a), blood (b), and bile (c) on T1-weighted spin echo MRI. Signal intensities of gadoterate, gadobenate, and gadoxetate at different concentrations in saline (d), blood (e), and bile (f) on T1-weighted turbo field echo MRI. *: significantly higher maximum signal intensity of the contrast agent than both other contrast agents; +: significantly higher maximum signal intensity than the contrast agent with the lowest maximum signal intensity. * or +: $p<0.05$, ** or ++: $p<0.01$; *** or +++: $p<0.001$. The data are extracted from Figure 2.

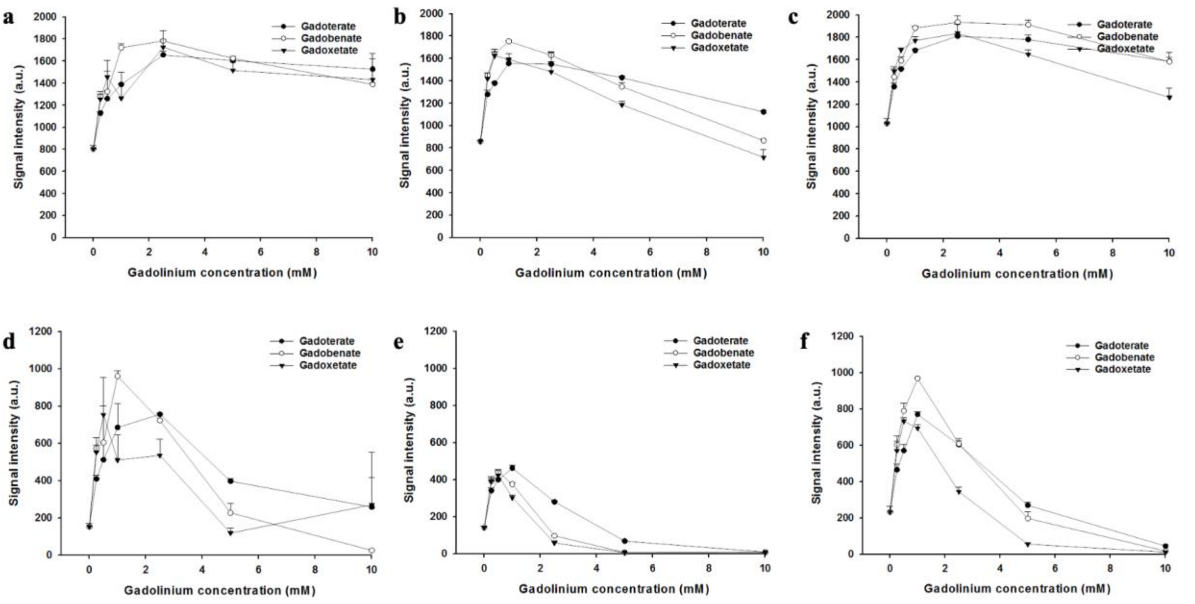

Supplementary Figure S3. Signal intensities of gadoterate, gadobenate, and gadoxetate in saline and biological fluids on 3D-mFFE and mGraSE MRI. Signal intensities of gadoterate, gadobenate, and gadoxetate at different concentrations in saline (a), blood (b), and bile (c) on 3D-mFFE MRI. Signal intensities of gadoterate, gadobenate, and gadoxetate at different concentrations in saline (d), blood (e), and bile (f) on mGraSE MRI. The data are extracted from Figure 3.

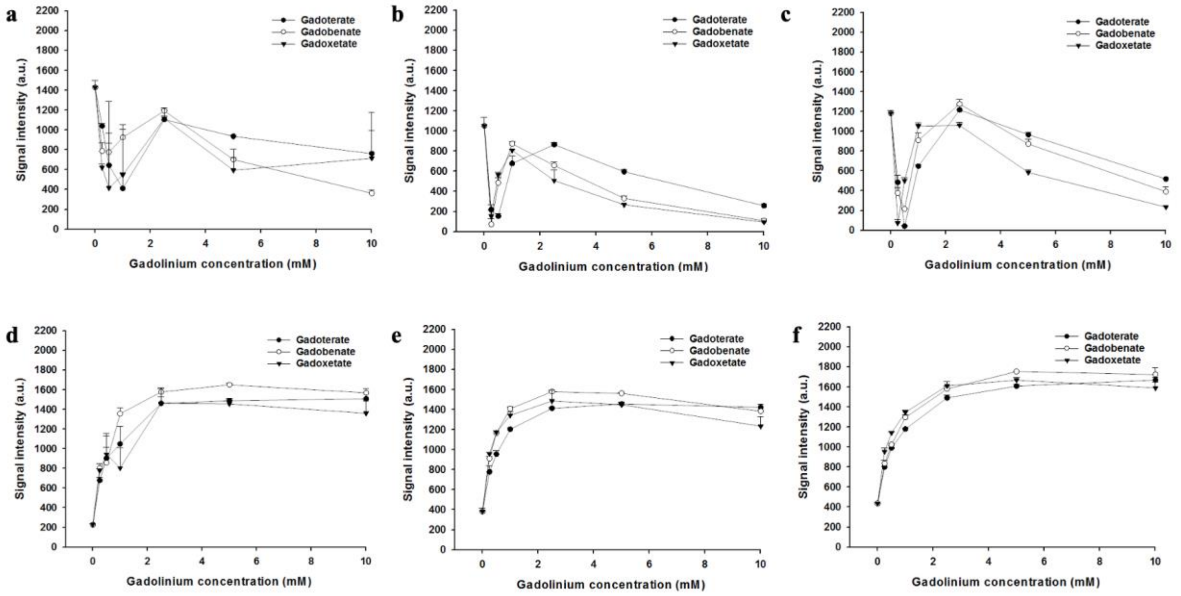

Supplementary Figure S4. Signal intensities of gadoterate, gadobenate, and gadoxetate in saline and biological fluids on STIR and eTHRIVE MRI. Signal intensities of gadoterate, gadobenate, and gadoxetate at different concentrations in saline (a), blood (b), and bile (c) on STIR MRI. Signal intensities of gadoterate, gadobenate, and gadoxetate at different concentrations in saline (d), blood (e), and bile (f) on E-THRIVE MRI. The data are extracted from Figure 4.

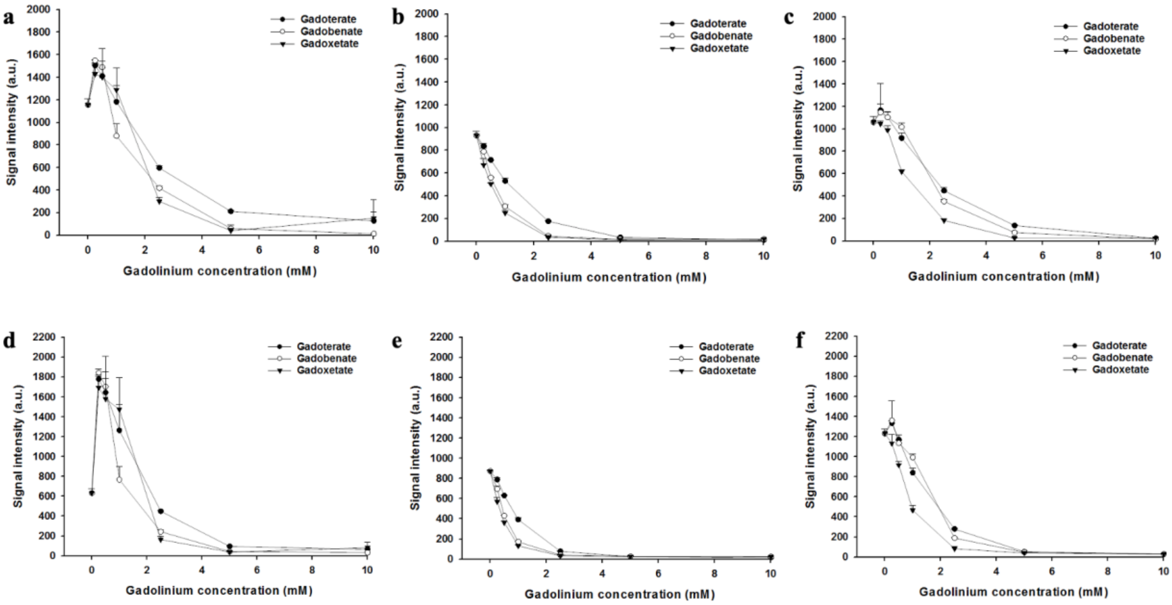

Supplementary Figure S5. Signal intensities of gadoterate, gadobenate, and gadoxetate in saline and biological fluids on T2-weighted TSE and FLAIR MRI. Signal intensities of gadoterate, gadobenate, and gadoxetate at different concentrations in saline (a), blood (b), and bile (c) on T2-weighted TSE MRI. Signal intensities of gadoterate, gadobenate, and gadoxetate at different concentrations in saline (d), blood (e), and bile (f) on FLAIR MRI. The data are extracted from Figure 6.

Supplementary Table S1. Summary table of main results in the comparisons of maximum signal intensities of contrast agents.

T1-weighted sequences	Saline	Blood	Bile
SE	Gadobenate > gadoterate, gadoxetate	Gadobenate > gadoterate, gadoxetate	Gadobenate > gadoterate > gadoxetate
TFE	No differences	Gadobenate, gadoxetate > gadoterate	Gadobenate > gadoterate
3D-mFFE	No differences	Gadobenate > gadoxetate, gadoterate	Gadobenate > gadoterate
mGraSE	No differences	No differences	Gadobenate > gadoterate > gadoxetate
STIR	No differences	Gadobenate, gadoterate > gadoxetate	Gadobenate, gadoterate > gadoxetate
eTHRIVE	Gadobenate > gadoterate, gadoxetate	Gadobenate, gadoxetate > gadoterate	Gadobenate > gadoxetate, gadoterate
**T2-weighted sequences**			
TSE	No differences	Gadobenate, gadoterate > gadoxetate	Gadobenate > gadoxetate
FLAIR	No differences	gadoterate > gadobenate > gadoxetate	No differences
